# Choline Regulates SOX4 through miR-129-5p and Modifies H3K27me3 in the Developing Cortex

**DOI:** 10.3390/nu15122774

**Published:** 2023-06-16

**Authors:** Evan M. Paules, Jorge A. Silva-Gomez, Walter B. Friday, Steve H. Zeisel, Isis Trujillo-Gonzalez

**Affiliations:** 1 Department of Nutrition, Gillings School of Global Public Health, University of North Carolina at Chapel Hill, Chapel Hill, NC 27514, USA; paulese@unc.edu (E.M.P.); jorgesil@unc.edu (J.A.S.-G.); walter_friday@unc.edu (W.B.F.); steven_zeisel@unc.edu (S.H.Z.); 2 Nutrition Research Institute, University of North Carolina at Chapel Hill, Kannapolis, NC 28081, USA

**Keywords:** choline, SOX4, EZH2, miR-125-9p, maternal nutrition, H3K27me3

## Abstract

Choline availability regulates neural progenitor cell proliferation and differentiation in the developing cerebral cortex. Here, we investigated the molecular mechanism underlying this process and demonstrated that choline regulates the transcription factor SOX4 in neural progenitor cells. Specifically, we found that low choline intake during neurogenesis reduces SOX4 protein levels, causing the downregulation of EZH2, a histone methyltransferase. Importantly, we demonstrate that low choline is not involved in SOX4 protein degradation rate and established that protein reduction is caused by aberrant expression of a microRNA (miR-129-5p). To confirm the role of miR-129-5p, we conducted gain-of-function and loss-of-function assays in neural progenitor cells and demonstrated that directly altering miR-129-5p levels could affect SOX4 protein levels. We also observed that the reduction in SOX4 and EZH2 led to decreased global levels of H3K27me3 in the developing cortex, contributing to reduced proliferation and precocious differentiation. For the first time, to our knowledge, we demonstrate that a nutrient, choline, regulates a master transcription factor and its downstream targets, providing a novel insight into the role of choline in brain development.

## 1. Introduction

Choline is an essential nutrient required for several metabolic functions through its different metabolites [1]. Phosphatidylcholine is an extensive component of cell membranes [2] and is also a precursor for the neurotransmitter acetylcholine. Choline is considered a primary methyl group donor through the production of betaine, which is used as a substrate to generate S-adenosylmethionine [3]. Choline-derived methyl groups are used in epigenetic regulation via DNA and histone methylation.

Choline can be made through de novo synthesis, requiring the methylation of phosphatidylethanolamine (catalyzed by the enzyme phosphatidylethanolamine N-methyl transferase (PEMT)) [4,5]. However, the de novo synthesis of choline is not sufficient to meet the requirements for all its biological functions; hence, it needs to be obtained from the diet [6]. Dietary choline intake is below the recommended intake levels in the US and developing countries [7,8,9]. This is particularly important for pregnant women since choline supply to the fetus is critical for the development of the central nervous system.

In rodents, low choline availability over the course of pregnancy results in spatial memory and memory processing deficits in the offspring [10,11,12]. Choline regulates neurogenesis by maintaining neural progenitor cell (NPC) self-renewal capacity [13,14], thereby impacting NPC cell fate. Cell fate—the decision to self-renew or differentiate—is critical for brain development and can be influenced by nutrient availability [15,16]. In addition, low choline (LC) status impacts brain development through the reduction in angiogenesis in the hippocampus, altering the cerebral cortex cytoarchitecture and reducing NPC proliferative capacity [14,17,18]. Specifically, under LC status, NPCs have an increased expression of microRNA-129-5p (miR-129-5p), resulting in a reduction in epidermal growth factor receptor (EGFR) protein levels and a subsequent decrease in NPC self-renewal [19]. Due to the reduction in NPC proliferative capacity, it is suggested that NPC cell fate would be biased towards premature differentiation. We specifically showed, in a mouse model, that when there is LC during neurogenesis (Embryonic days 11.5–17.5), the numbers of early-born neurons are increased and late-born neurons are decreased [14], reinforcing the notion that an increase in miR-129-5p may induce precocious NPCs differentiation.

SRY-box transcription factor 4 (SOX4) is a master transcription factor that plays a significant role in organogenesis, development, and chromatin structure [20,21]. An aberrant expression of SOX4 is present in different cancer types, including melanoma, which promotes cell migration and invasion [22]. Furthermore, in colorectal and breast cancer, SOX4 expression is dysregulated, in part, by a reduced expression of miR-129-5p [23,24]. In the developing brain, SOX4 is critical for neurogenesis in that it maintains the self-renewal capacity of NPC and intermediate progenitor cell (IPC) phenotypes [25]. SOX4 acts, in part, as a molecular switch, whereby the differentiation of NPCs and IPCs can be effectively executed when SOX4 is reduced [25,26]. This molecular switch occurs within the ventricular and subventricular zones of the cerebral cortex, at which point the NPCs and IPCs migrate and differentiate [25,26]. However, the regulation of SOX4 in the developing brain by nutrient availability is unknown.

In this study, we determined that NPCs with an LC status have reduced SOX4 protein levels. The reduction in SOX4 protein was mediated by increased levels of miR-129-5p and the binding of miR-129-5p to *Sox4* 3′UTR, not to the increased degradation of SOX4. Moreover, we found that SOX4 regulates the expression of enhancer zeste homolog 2 (EZH2) in NPCs. EZH2 is a methyltransferase that modifies H3 histones at lysine 27 (H3K27me3), thereby controlling the balance between the self-renewal and differentiation of NPCs in the cerebral cortex [27,28,29]. NPCs with an LC status have reduced expression of EZH2, demonstrating a reduction in SOX4 transcriptional output, which corresponds with its lower protein levels in the nucleus. Additionally, we confirmed a specific reduction in H3K27me3 in the cerebral cortex of LC fetuses. These findings provide additional insights through which choline influences microRNAs, transcription factor expression, and ultimately brain development.

## 2. Materials and Methods

### 2.1. Animals

Nestin-CFPnuc transgenic mice were generously provided by Dr. Grigori Enikolopov (Cold Spring Harbor Laboratory, Cold Spring Harbor, NY, USA), and were maintained in a C57BL/6J (The Jackson laboratory) background. All mice were bred and maintained at the David H. Murdock Research Institute (DHMRI), Center for Laboratory Animal Science Facilities. All animal protocols were approved by the David H. Murdock Research Institute, Institutional Animal Care and Use Committee (IACUC). Genotyping was performed according to the providers’ protocols [30]. Mice were kept in a temperature control environment at 24 °C and exposed to 12 h light and dark cycles.

### 2.2. Diets

All animals were maintained on a medium choline diet (MC; modified AIN93G diet with 1.4 g/kg choline chloride; Research Diets Inc., New Brunswick, NJ, USA; cat# D16040703, Table S1) for at least two weeks before mating. Following timed mating, all dams remained on the MC diet until E11.5. At E11.5, the time-mated dams were randomly assigned one of three feeding groups: low choline (LC; modified AIN93G diet with 0 g/kg choline chloride D16040705; see Table S1), MC, or high choline (HC; modified AIN93G diet with 6.3 g/kg choline chloride D16040706; see Table S1). Mice remained in the assigned diets until E17.5.

### 2.3. In Vitro Neural Progenitor Cell Culture

NPCs were isolated from E14.5 cerebral cortices as previously described [19]. In brief, E14.5 cerebral cortices were collected, originating from different dams, and dissociated into a single-cell suspension using Accutase (Thermo, Waltham, MA, USA; cat# 00-4555-56). The cells were maintained in a neurobasal media (Gibco, Waltham, MA, USA; cat# 21103049) supplemented with a B27 supplement without vitamin A (Gibco, cat# 1087889), recombinant human fibroblast growth factor (Gibco, cat# PHG0023), and recombinant human epidermal growth factor (Gibco, cat# PHG0313). Cells derived from male and female embryos were kept as separate cultures and remained separate for all experiments. All experiments were conducted in custom choline-free neurobasal media, supplemented with 2 Mm L-glutamine (Gibco, cat# 25030149), recombinant human fibroblast growth factor (Gibco, cat# PHG0023), recombinant human epidermal growth factor (Gibco, cat# PHG0313), and B27 supplement without vitamin A (Gibco, cat# 1087889). A choline chloride (Millipore Sigma, Burlington, MA, USA; cat# C7017) solution was made with phosphate buffer saline, which was filter sterilized and added to the choline-free custom neurobasal media to achieve the following final concentrations: LC (5 μm), MC (70 μm), and HC (315 μm). These choline concentrations are based on choline metabolites quantified in mice tissues and choline dose-response curves in NPCs, which were performed in our laboratory in conjunction with our published data [14,17,19,31,32]. To expand the NPC culture, NPCs were maintained in a proliferative, neurosphere culture and attachment was avoided by gently rocking the plates every day. The neurospheres were expanded over 4 passages and all the experiments were conducted at passage #5, with the NPCs grown in a monolayer in either 6-well plates (Celltreat, Pepperell, MA, USA; cat# 229105) or 24-well plates (Corning, Glendale, AZ, USA; cat# 3524), precoated with poly-ornithine (Sigma, cat# A-004-C) and fibronectin (Millipore Sigma, cat# F1141).

### 2.4. Cycloheximide Treatment in Cultured Neural Progenitor Cells

NPCs were isolated and cultured as described above. NPCs were plated and treated for 24 h with a custom choline-free media, as described prior, with varying concentrations of choline chloride: LC (5 μm), MC (70 μm), and HC (315 μm). At 24 h, NPCs were treated with 540 nm cycloheximide (Selleck chemical, Houston, TX, USA; cat# S7418) and collected at the following time points: 0, 15 min, 30 min, 45 min, and 60 min. For collection, NPCs were washed using phosphate buffer saline and chemically dissociated using TrypLE (Thermo, cat# 12605010). The cells were then transferred to a 1.5 mL Eppendorf tube and pelleted at 4 °C via centrifugation, flash-frozen in liquid nitrogen, and stored at −80 °C.

### 2.5. Western Blot Analysis

Whole-cell lysates were prepared using RIPA buffer with added protease (Sigma, St. Louis, MO, USA; cat# 05892970001) and phosphatase inhibitor cocktails (Sigma, cat# 4906845001) for in vitro and in vivo experiments. Following the addition of the RIPA buffer, the whole-cell lysates were sonicated for 5 min. Protein quantification for all samples was conducted using a Bradford assay (Bio-Rad, Hercules, CA, USA; cat# 5000006) following the manufactures protocol. Laemmli buffer was added to each protein sample followed by boiling, for 5 min, using a heating block. Protein sample preparation for nuclear fractionation experiments were conducted using the Abcam nuclear extraction kit (Abcam, Cambridge, UK; cat# ab113474) and histone extraction were conducted using the Histone Extraction Kit (Abcam, Cambridge, UK; cat# ab113476). Proteins were loaded into SDS-PAGE gels and blotted on nitrocellulose membranes (Cytiva, Marlborough, MA, USA; cat# 10600003). Prior to immunolabeling, the nitrocellulose membranes were treated with Revert 700 total protein stain (LI-COR, Lincoln, NE, USA; cat# 926-11010) following the manufacturers protocol, to assess successful protein transfer. Following the total protein stain protocol, nitrocellulose membranes were blocked in a 5% bovine serum albumin in a tris-buffered saline (0.1% tween-20) solution for 1 h. Immunolabelling was accomplished with the following antibodies: SOX4 1:500 (Santacruz, Dallas, TX, USA; cat# sc-518016), SDCBP 1:500 (Santacruz, cat# sc-515538), EZH2 1:1000 (Abcam cat# ab186006), Alpha Tubulin 1:1000 (Santacruz, cat# sc-8035), Lamin B1 1:1000 (Abcam, cat# ab16048), H3K27me3 1:1000 (Abcam, cat#6002), H3K9me3 1:1000 (Abcam, cat#8898) and total Histone 3 (H3) 1:1000 (Cell Signaling, cat# D1GH2). All antibodies were diluted in the 5% bovine serum albumin blocking solution, as described prior, and the membranes were incubated in their respective antibody overnight. Secondary antibodies were goat anti-rabbit 1:1000 (LI-COR, cat# 926-68071), anti-mouse fluorescent antibodies 1:1000 (LI-COR, cat# 926-32210), or anti-chicken (LI-COR, cat# 926-32218). The membranes were imaged in a LICOR Odyssey^®^ imaging system.

### 2.6. miR Bioinformatics

miR bioinformatics were carried out using two different databases: TargetScan and miRDB [33,34,35]. TargetScan (http://www.targetscan.org; accessed on 11 December 2018) predicts binding patterns with miRs of interest using a few different parameters, including site type, supplementary pairing, local AU, minimum distance, 3′ untranslated region, and others. These parameters are scored and aggregated into a score denoted as a context++ score. The more negative the context++ score is, the greater the probability of the miR targeting the gene product of interest. MiRDB (http://mirdb.org; accessed on 11 December 2018) uses a similar approach via their target score. The target score is a composition of miRNA overexpression and subsequent RNA-sequencing data in conjunction with public CLIP-sequencing data, which are funneled into a support vector machine that identifies similarities between miR-binding to 3′-untranslated regions of mRNA and the subsequent downregulation of pathways associated with the mRNA in question. The closer the target score is to 100, the greater the probability that the miR of interest binds to a respective mRNA.

### 2.7. RT-PCR Analysis

Total RNA was isolated from in vitro NPCs, treated with either LC, MC, or HC, using an miRNeasy mini kit (Invitrogen, Waltham, MA, USA; cat# 12183018A), while total RNA from cerebral cortices was isolated using an RNeasy Plus Universal mini kit (Qiagen, Hilden, Germany; cat# 73404), both of which were used following the manufacturers’ protocols. For mRNA, complementary DNA (cDNA) synthesis was completed using an Applied Biosystems High-Capacity cDNA Reverse Transcription kit (Thermo, cat# 4368814) following the manufacturers’ instructions. Reverse transcriptase polymerase chain reaction (RT-PCR) was conducted using SsoAdvanced Universal SYBR Green Supermix (Biorad Laboratories, Hercules, CA, USA; cat# 1725274) following the manufacturers’ protocol. All experiments were run on a Roche Light 480 (Roche Applied Science, Mannhelm, Germany). The primers used include *Sox4, Sdcbp, Ezh2* and TATA box binding protein (*Tbp*) (for sequences please see Table S2) [36,37]. miR RT-PCR was conducted by isolating RNA, as described above, and subsequently using Applied Biosystems reverse transcriptase for miRs (Thermo, cat# 4366596), following the manufacturers’ protocol. This was completed in conjunction with Taqman primers for the following miRs and normalizers: miR-129-5p and small nucleolar RNAs (SNORD)-234. Values for both mRNA and miR RT-PCR were normalized using the delta cycle time method [38].

### 2.8. Transfection of Neural Progenitor Cells

Neural progenitor cells were plated as monolayers (as described above) and were transfected after 24 h, as previously described [19]. Briefly, cells were transfected using Lipofectamine RNAiMAX (Life technologies, cat#13778-150) with miR-129-5p mimics (Invitrogen, cat#4464066) or scrambled control (Invitrogen, cat#4464058). For experiments where we inhibited miR-129-5p, NPCs were transfected with locked nucleic acid-modified oligonucleotide miR-129-5p inhibitor (Qiagen, cat# Y104102971-DDC) or scrambled inhibitor control (Qiagen, cat# Y100199006-DDB).

### 2.9. miR-Pulldown

To test in NPCs if miR-129-5p were binding to the 3′UTR of *Sox4*, we performed a targeted capture experiment, as previously described [39,40]. Briefly, neural progenitor cells were transfected with biotin-tagged miR-129-5p or biotin-tagged scramble miR duplexes using Lipofectamine RNAiMAX (Life technologies, cat#13778-150) according to the manufacturer’s protocol. After 24 h, cells were harvested and lysed in a 10 mM KCl, 1.5 mM MgCl2, 60 U/mL RnaseOut, 0.5% IGEPAL CA-630, 5 mM DTT, and 1 × Complete Mini EDTA-free protease inhibitor cocktail. Samples were frozen on dry ice and thawed at room temperature (22 °C), followed by centrifugation at 13,000 rpm at 4 °C for 2 min. The cleared lysates were transferred to a new tube and NaCl was added to a final concentration of 1M. Samples were combined with Dynabeads MyOne Streptavidin (Invitrogen, cat# 650-01); the beads were previously blocked overnight with 1 µg/µL BSA, 1 µg/µL Yeast tRNA, and put on a rotating mixer at room temperature (22 °C) for 30 min. After incubation, Dynabeads were washed three times with wash buffer: 10 mM Tris-Cl pH 7.5, 10 mM KCl, 1.5 mM MgCl2, 0.5% IGEPAL CA-630, 1 M NaCl, 5 mM DTT, 60 U/mL RnaseOut, and 1 × Complete Mini EDTA-free protease inhibitor cocktail, to remove molecules non-specifically bound to the beads.

After washing, beads were resuspended in 100 μL of RNAse-/DNAse-free water and total RNA was purified using the Pure Link RNA Mini Kit (Invitrogen cat#12183018A) according to the manufacturer’s instructions. Target transcript *Sox4* was evaluated by qPCR as described above.

### 2.10. Targeted NextGen Bisulfite Sequencing for the miR-129 Gene

Neural progenitor cells were plated as monolayers and treated with LC (5 μm) and MC (70 μm) for 48 h. Cells were harvested and kept at −80 °C until processing. NPCs Digestion: Cell pellets were lysed using M-digesting Buffer (ZymoResearch; Irvine, Ca; cat# D5021-9) and 5–10 μL of protease K (20 mg/mL), with a final M-digestion concentration of 2X. The samples were incubated at 65 °C for at least 2 h. Bisulfite Modification: 20 μL of supernatant from the sample’s extracts was bisulfite-modified using the EZ-96 DNA Methylation-Direct Kit (ZymoResearch; Irvine, CA, USA; cat# D5023) as per the manufacturer’s instructions. The bisulfite-modified DNA samples were eluted using M-elution Buffer in 46 μL. Multiplex PCR: All bisulfite-modified DNA samples were amplified using separate multiplex or simplex PCRs. PCRs included 0.5 units of HotStarTaq (Qiagen; Hilden, Germany; cat# 203205), 0.2 µM primers, and 3 µL of bisulfite-treated DNA in a 20 µL reaction. All PCR products were verified using the Qiagen QIAxcel Advanced System (v1.0.6). Prior to library preparation, PCR products from the same sample were pooled and then purified using the QIAquick PCR Purification Kit columns or plates (cat# 28106). Samples were run alongside established reference DNA samples with a range of methylations. They were created by mixing high- and low-methylated DNA to obtain samples with 0, 5, 10, 25, 50, 75, and 100% methylation [41]. The high-methylated DNA is in vitro enzymatically methylated genomic DNA with >85% methylation. The low-methylated DNA is chemically and enzymatically treated with <5% methylation. Library Preparation and Sequencing Libraries: We used a custom library preparation method created by EpigenDx. Next, library molecules were purified using Agencourt AMPure XP beads (Beckman Coulter; Brea, CA, USA; cat# A63882). Barcoded samples were then pooled in an equimolar fashion before template preparation and enrichment were performed on the Ion Chef™ system using Ion 520™ and Ion 530™ ExT Chef reagents (Thermo Fisher; Waltham, MA, USA; cat# A30670). Following this, enriched, template-positive library molecules were sequenced on the Ion S5™ sequencer using an Ion 530™ sequencing chip (cat# A27764). Data Analysis: FASTQ files from the Ion Torrent S5 server were aligned to a local reference database using the open-source Bismark Bisulfite Read Mapper program (v0.12.2) with the Bowtie2 alignment algorithm (v2.2.3). Methylation levels were calculated in Bismark by dividing the number of methylated reads by the total number of reads. An R-squared value (RSQ) was calculated from the controls set at known methylation levels, to test for PCR bias.

### 2.11. Immunofluorescence, Microscopy, and Data Analysis

Immunofluorescence was conducted as previously described [19]. In brief, E17.5 brains were fixed in 4% paraformaldehyde, cryoprotected in a sucrose gradient from 10–30%, and subsequently frozen. Coronal sections (20 μm) via cryosectioning were prepared for immunostaining with a blocking solution constituting of 5% goat serum and 0.1% TritionX-100. The coronal sections were subsequently incubated with the following antibodies: SOX4 1:500 (Santa Cruz, Dallas, TX, USA; cat# sc-518016); H3K27me3 1:1000 (Cell Signaling, Boston, MA; cat# C36B11) and RNA pol-II 1:500 (Abcam, cat# ab252855). The antibodies were diluted in the blocking solution and incubated overnight. The next day, slides were washed with blocking solution and incubated with the secondary antibodies, which included goat anti-mouse Alexa-Fluor 488 1:500 (Jackson ImmunoResearch, West Grove, PA, USA; cat# 111-545-003), goat anti-rabbit Alexa-Fluor 488 1:1000 (Jackson ImmunoResearch, West Grove, PA, USA; cat# A11034), goat anti-mouse Alexa-Fluor 555 1:1000 (Jackson ImmunoResearch, West Grove, PA, USA; cat# A21434), and 4′,6-diamidino-2-phenylindole (DAPI) 1:2000 (Thermo, cat# D1306), which were all diluted in the blocking solution for 1 h. Following incubation, slides were washed with the blocking solution and mounted using fluormount (Sigma; cat# F4680). Images were captured using a Zeiss LSM 710 confocal microscope (Zeiss GmbH, Oberkochen, Germany). Z-stacks were acquired for each coronal section with 40× objectives. Cell counts and tissue measurements were obtained with ImageJ version 1.54c (NIH; Bethesda, MD, USA) and LSM Browser (Zeiss GmbH) software, version 4.2.0.121. All quantifications were blinded to the Research Technician.

### 2.12. Statistical Analyses

Statistical analyses were performed using Prism 9 (GraphPad Software, La Jolla, CA, USA) and power analysis was conducted using G*Power 3 [42]. The distribution of data was tested for normality and the Brown–Forsythe test (F test) was used to compare variances amongst experimental groups. Outliers were determined using ROUT test with a Q = 1%. Groups that had non-significant variances were compared using One-way ANOVA or *t*-test. Groups with significant variances were compared using non-parametric Kruskal–Wallis test or Mann–Whitney test. Data are presented as mean ± standard error.

## 3. Results

### 3.1. miR-129-5p Is Predicted to Bind to SOX4 mRNA and SOX4 Protein Is Reduced in the SVZ of E17.5 Cortices under an LC Status

**Figure 1:**
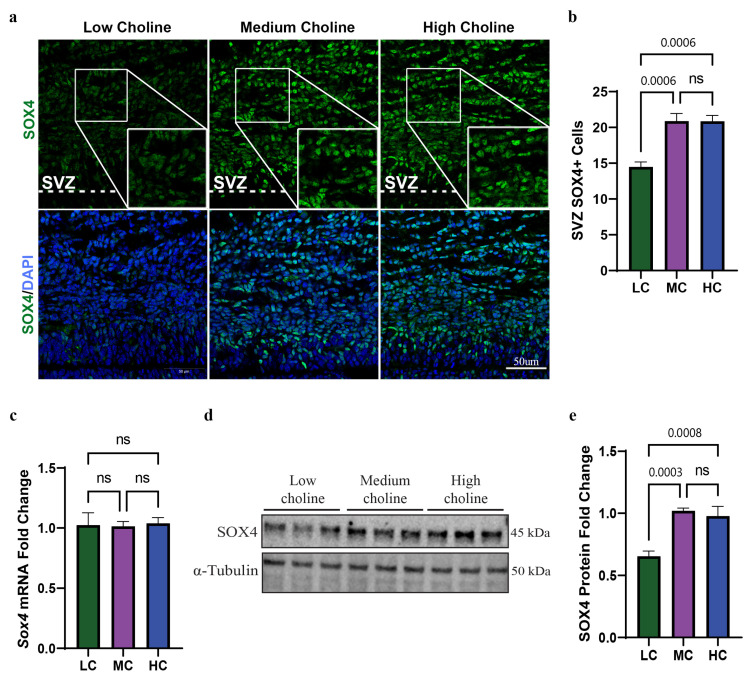
Low choline availability results in reduced SOX4 protein levels in NPCs isolated from E17.5 brains and in fetal cerebral cortex. Time-mated dams were placed on either a low, medium, or high choline diet from E11.5 to E17.5. (**a**) The quantification of the SVZ in E17.5 embryos showed a reduction in SOX4-positive cells under low-choline conditions (*n* = 5 dams per condition, 2 pups per dam) (*p* = 0.0006, ns = not significant). (**b**) Immunostaining was used to visualize the cells of the SVZ under low-choline conditions, showing reduced numbers of SOX4-positive (seen in green) compared to cells under LC, MC, or HC conditions (*n* = 5 dams per condition, 2 pups per dam) (scale bar: 50 μm). (**c**) mRNA levels of *Sox4* in NPCs were determined using RT-PCR and demonstrated no change in *Sox4* mRNA under any choline condition (*n* = 5 dams per condition, 1–4 pups per dam) (ns = not significant). (**d**) Quantification of SOX4 shows a reduction in protein levels in NPCs under low-choline conditions relative to medium- and high-choline conditions (*n* = 6 dams per condition, 1–4 pups per dam) conditions (HC vs. LC *p* = 0.0008, MC vs. LC *p* = 0.0003, ns = not significant). (**e**) Representative Western blot of SOX4 expression LC, MC, and HC conditions, showing a reduction in SOX4 in LC. Data are mean ± SEM. All data were tested for normality by Brown–Forsythe test and analyzed by One-way ANOVA. LC = low choline, MC = medium choline.

We previously found in sorted NPCs that LC intake during neurogenesis led to the overexpression of miR-129-5p [19]. This miR is expressed primarily in the subventricular zone (SVZ) and ventricular zone (VZ) of the cortex over the course of neurogenesis, and its expression is elevated in the later stages of neurogenesis (E16.5–E17.5) [43]. Using the miR databases TargetScan and miRDB, we found that miR-129-5p was predicted to target *Sox4* in four different binding sites in the 3′ untranslated region (UTR) (Figure S1A). However, binding between miR-129-5p and *Sox4* had not been determined in NPCs. Since SOX4 is also expressed in the SVZ and, to a lesser degree, the VZ during the same period of neurogenesis as miR-129-5p, this suggested to us that miR-129-5p could bind to *Sox4* in NPCs [25]. First, we evaluated protein levels of SOX4 in the SVZ. To achieve this, we collected E17.5 cortices from dams fed either an LC, MC, or HC diet and subsequently conducted immunostaining for SOX4 protein in the SVZ of E17.5 fetuses. From the immunostainings, we found a reduction in SOX4-positive cells in the SVZ of the LC brains when compared with MC and HC, and no differences in SOX4-positive cells were observed between MC and HC. (Figure 1a,b). This led us to confirm that SOX4-positive cells are reduced under maternal low choline availability in the SVZ of the developing brain.

### 3.2. SOX4 Protein, but Not mRNA, Is Reduced in NPCs In Vitro at 48 h with an LC Status

We reasoned that reduction in SOX4-positive cells in the SVZ was probably driven by its low protein levels in NPCs in LC condition. To test this, we used an in vitro model by culturing NPCs in either LC, MC, or HC media. We initially used qPCR to assess mRNA levels of *Sox4* and found no changes in its mRNA under any choline condition at 48 h (Figure 1c). However, we found around a ~40% reduction in SOX4 protein level by Western blot in NPCs under LC availability for 48 h (Figure 1d,e). This further suggested to us that *Sox4* was prospectively targeted post-transcriptionally via miR-129-5p. This also recapitulates our findings in vivo, validating our in vitro model. Since we found no differences between MC and HC in *Sox4* mRNA or SOX4 protein expression, this substantiated to us that MC was a sufficient level of choline to compare against low choline availability to test the rest of our hypothesis. Together, these results demonstrated that LC availability in vivo and in vitro significantly reduces the protein levels of SOX4 in NPCs.

### 3.3. SOX4 Protein Is Reduced in Both the Cytoplasm and Nucleus In Vitro in NPCs with an LC Status

**Figure 2:**
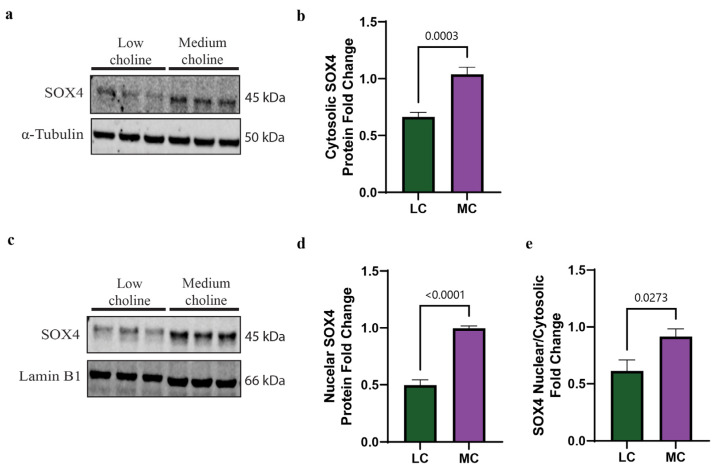
SOX4 protein levels are reduced in both cytoplasm and nucleus in vitro under low-choline conditions. Cortical NPCs were cultured in either LC (5 μM) or MC (70 μM) for 48 h. (**a**) Representative Western blot of cytosolic SOX4 protein expression in LC and MC conditions. (**b**) Quantification of SOX4 cytosolic protein levels shows a reduction in SOX4 in NPCs under low-choline conditions compared to MC (cells derived from *n* = 5–6 dams per condition, 1–4 pups per dam) (*p* = 0.0003). (**c**) Representative Western blot of nuclear SOX4 protein expression in LC and MC conditions. (**d**) SOX4 protein quantification shows a reduction in SOX4 in the nucleus of NPCs under LC conditions compared to MC, suggesting a reduction in SOX4 DNA binding capacity (cells derived from *n* = 5–6 dams per condition, 1–4 pups per dam) (*p* < 0.0001). (**e**) SOX4 nuclear cytosolic ratio is overall reduced under LC conditions (cells derived from *n* = 5–6 dams per condition, 1–4 pups per dam) (*p* = 0.0273). Data are mean ± SEM. All data were tested for normality by Brown–Forsythe test and analyzed by an unpaired *t*-test. LC = low choline, MC = medium choline.

One of the regulation steps of SOX transcription factors is the trafficking of the proteins between cytoplasm and nucleus to balance the access to their target genes [44]. We wondered if the reduction in SOX4 protein levels in NPCs was specific to a cellular compartment. To test this, we treated NPCs with LC or MC media for 48 h, followed by cellular fractionation to separate cytoplasmic and nuclear SOX4 protein. Corresponding protein levels were then determined through Western blot. We found a statistically significant reduction in SOX4 in the cytoplasm of NPCs treated with LC, of ~40% (Figure 2a,b). When we evaluated protein levels in the nucleus of NPCs, we found a more pronounced reduction in SOX4, showing a ~50% decrease (Figure 2c,d). Moreover, the ratio between nuclear and cytosolic SOX4 showed a significant reduction in SOX4 protein levels under LC conditions (Figure 2e). Since SOX4 is a transcription factor that regulates many developmental processes [45], our results strongly suggest that a reduction in SOX4 in the nucleus could have an impact on their target genes, thus altering the cell fate of NPCs.

### 3.4. EZH2 Protein and mRNA Are Reduced In Vitro in NPCs with an LC Status

**Figure 3:**
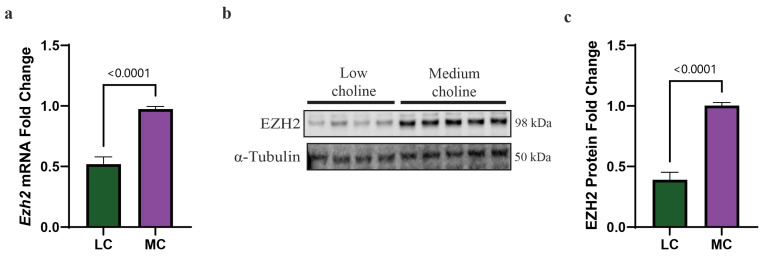
Low choline results in reduced expression of the SOX4 target *Ezh2* at mRNA and protein levels, in NPCs. Cortical NPCs were cultured in either low choline (5 μM) or medium choline (70 μM) for 48 h. (**a**) mRNA levels of *Ezh2* are reduced under LC conditions compared to MC conditions demonstrating that SOX4 reduction has transcriptional effects on one of its targets (cells derived from *n* = 6 dams per condition, 1–4 pups per dam) (*p* < 0.0001). (**b**) Quantification of EZH2 protein levels is also reduced under low-choline conditions compared to medium choline, illustrating the downstream consequences of reduced SOX4 transcriptional outputs (cells derived from *n* = 7 dams per condition, 1–4 pups per dam) (*p* < 0.0001). (**c**) Representative Western blot of EZH2 protein under LC and MC conditions. Data are mean ± SEM. All data were tested for normality by Brown–Forsythe test and statistical analysis was performed using an unpaired *t*-test. LC = low choline, MC = medium choline.

SOX4 is a transcriptional factor that regulates genes involved in the cell cycle and cell migration. The promoter analysis of the SOX4 transcriptional network has been established in different cell types, including cancer and neural crest cells [46,47]. One of these target genes is enhancer of zeste homolog 2 (*Ezh2*). EZH2 is a major regulatory transcription factor that modulates NPC and IPC differentiation [27,28,48]. Together, SOX4 and EZH2 act as a molecular switch that facilitates the transition of NPCs and IPCs from progenitor cells to differentiated neurons [25,28,29]. To identify if the reduction in SOX4 we had found in NPCs treated with LC (5 µM) influenced this molecular switch, we sought to assess expression levels of EZH2. We first evaluated by qPCR the mRNA levels of *Ezh2* and found a 50% reduction in LC compared to MC (Figure 3a). Then, we performed a Western blot to assess protein levels of EZH2, and we observed a reduction of ~65% of EZH2 protein in NPCs exposed to LC (Figure 3b,c). The reduction in both SOX4 and EZH2 in NPCs, under LC conditions, suggested changes to the NPC cell fate. The reduction in this SOX4/EZH2 switch in NPCs potentially helps to explain, in part, earlier observations made by our lab showing that pups from dams fed an LC diet have a reduced number of late born neurons in the cortex of their brain, yet, also have an increased number of early born neurons at embryonic day 17.5 (E17.5), resulting in aberrant cortical layering [14].

### 3.5. SOX4 Is Not Degraded at an Increased Rate with an LC Status In Vitro

**Figure 4:**
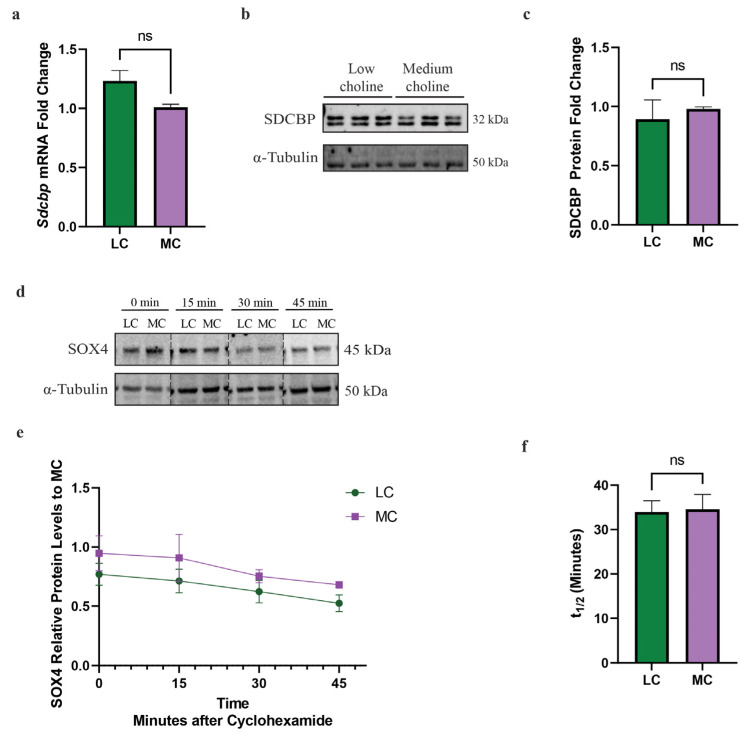
*Sdcbp* mRNA and protein are unchanged in NPCs in vitro. Cortical NPCs were cultured in either LC (5 μM) or MC (70 μM) for 48 h. For half-life and protein degradation rate assays, cortical NPCs were cultured in either LC (5 μM) or MC (70 μM) for 24 h and subsequently treated with cycloheximide. (**a**) mRNA levels of *Sdcbp* are unchanged under low-choline conditions (cells derived from *n* = 6 dams per condition, 1–4 pups per dam) (ns = not significant). (**b**) Protein levels of SDCBP, determined by Western blot, between LC and MC. (**c**) SDCBP protein quantification is also unchanged, suggesting SOX4 does not have increased degradation rates due to a reduction in its binding partner SDCBP (ns = not significant). (**d**) Degradation rate of SOX4 protein is unchanged between under low-choline conditions (cells derived from *n* = 3–4 dams per condition, 1–4 pups per dam). (**e**,**f**) Half-life of SOX4 degradation is unchanged under low-choline conditions, indicating SOX4 is not undergoing degradation more rapidly than medium choline; (cells derived from *n* = 3–4 dams per condition, 1–4 pups per dam) (ns = not significant). All data were tested for normality by Brown–Forsythe test and analyzed by analyzed by Mann–Whitney test (**a**,**c**) or unpaired *t*-test (**e**,**f**). LC = low choline, MC = medium choline.

After confirming that SOX4 protein levels are reduced in vivo and in vitro, and that a major transcriptional target of SOX4, EZH2, is reduced in NPCs, we sought to determine if there were other mechanisms involved in SOX4 protein regulation. The post-transcriptional regulation of SOX4 protein can be mediated via two possible mechanisms: (1) increased rate or altered degradation of SOX4 protein or (2) a post-transcriptional mechanism, such as miR-129-5p. Although both TargetScan and miRDB predicted the binding of miR-129-5p to *Sox4*, we wanted to determine if miR binding was the sole mechanism of SOX4 degradation in our NPC model. SOX4 post-transcriptional regulation is mediated by proteasomal degradation by a ubiquitin- independent mechanism through Proline (*p*), glutamic acid (E), serine (S) and threonine (PEST) motif [49]. This PEST region is located in the C-terminal of SOX4 and is targeted by the proteasome, resulting in its degradation [50]. Hence, in order for SOX4 to translocate to the nucleus, function as a transcription factor, and to prevent its degradation, it must bind to its primary binding partner syndecan binding protein (SDCBP) [50]. Due to the importance of SDCBP, we wanted to determine if a reduction in SDCBP was playing a role in the reduction in SOX4 protein. We found that at 48 h, with LC or MC, there were no significant differences in *Sdcbp* mRNA by qPCR (Figure 4a). Then, we proceeded to evaluate protein levels of SDCBP, by Western blot, between LC and MC status and did not find significant differences (Figure 4b,c). This suggested that the lack of a primary SOX4 binding partner is not contributing to the mechanism by which SOX4 is reduced in NPCs.

Alternatively, we hypothesized that the proteasomal degradation rate was increased and, therefore, responsible for the reduction in SOX4 in NPCs with an LC status. To test this hypothesis, we treated NPCs with cycloheximide, a ribosomal inhibitor that blocks the translation of mRNA to protein, to determine the subsequent rate of SOX4 degradation over time. We found that SOX4 rapidly degraded and is not apparent after 45 min, matching previous findings [50]. Moreover, we found no differences in the rate of degradation between LC and MC status (Figure 4d,e). The half-life for SOX4 was also found not to be statistically significant between LC and MC status (Figure 4f). Since there was no change to the SDCBP protein nor translation rate of SOX4, we concluded that the degradation rate of SOX4 did not play a role in the reduction of its protein levels. Taken together, these data demonstrate that a reduction in SOX4 protein in NPCs is not mediated by proteasomal degradation. These findings then led us to further investigate the potential role of miR-129-5p in the reduction in SOX4 protein.

### 3.6. MiR-129-5p Is Increased in NPCs with an LC Status In Vitro and Targets SOX4 mRNA

**Figure 5:**
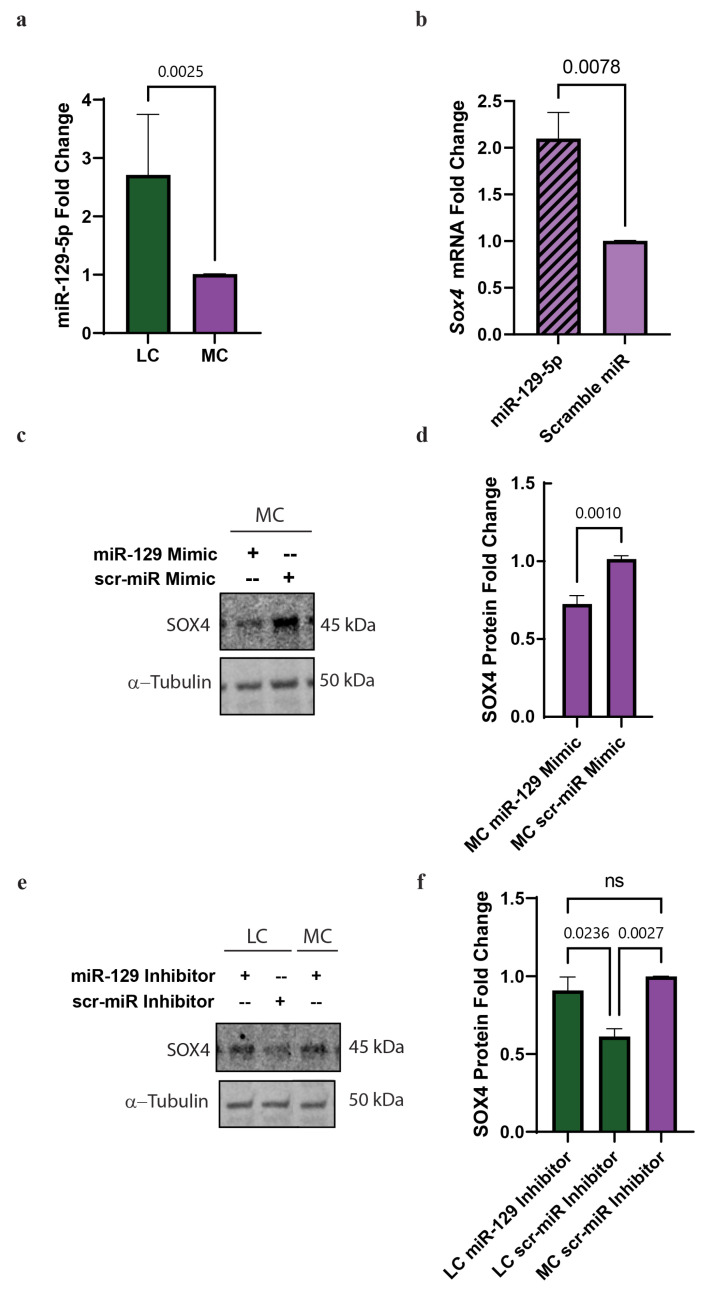
miR-129-5p is upregulated in low choline and binds to *Sox4* 3′UTR in NPCs with an LC status in vitro. Cortical NPCs were cultured in either LC (5 μM) or MC (70 μM) for 48 h. (**a**) miR-129-5p is upregulated in NPCs under LC conditions (cells derived from *n* = 5 dams per condition, 1-4 pups per dam) (*p* = 0.0025). (**b**) *SOX4* is pulled-down with miR-129-5p demonstrating miR-129-5p binding to SOX4 (cells derived from *n* = 4 dams per condition, 1–4 pups per dam) (miR-129-5p vs. scramble miR *p* = 0.0024, miR-129-5p vs. mock *p* < 0.0001, scramble miR vs. mock *p* = 0.0059). (**c**) Transfections of a miR-129-5p mimic on NPCs under MC conditions reduced the presence of SOX4 protein (cells derived from *n* = 4–5 dams per condition, 1–4 pups per dam) (*p* = 0.001). (**d**) miR-129-5p inhibitor resulted in the inability for miR-129-5p to bind to *SOX4* mRNA, resulting in the increase in SOX4 protein (cells derived from *n* = 5 dams per condition, 1–4 pups per dam) (*p* = 0.0178). Data mean ± SEM. (**e**) SOX4 expression from NPCs transfected with either miR-129-5p inhibitor or scramble miR inhibitor and treated the cells with MC or LC and (**f**) Quantification of SOX4 fold change in the NPCs transfected with either miR-129-5p inhibitor or scramble miR inhibitor. All data were tested for normality by Brown–Forsythe test and analyzed by analyzed by Mann–Whitney test (**a**) unpaired *t*-test (**b**,**d**), or One-way ANOVA (**f**). LC = low choline, MC = medium choline.

Our second hypothesis for the observed reduction in SOX4 protein was that the *Sox4* 3′UTR was targeted by a post-transcriptional mechanism, thereby inhibiting the translation of *Sox4* mRNA while not leading to a reduction in its RNA levels in the NPCs. Since we had predicted miR-129-5p to bind to *Sox4* mRNA, we wanted to determine that miR-129-5p was increased in our in vitro model. Using qRT-PCR, we found that miR-129-5p was increased ~2.5-fold in NPC treated with LC in vitro (Figure 5a).

Next, we tested if we could detect the binding of mir-129-5p to the *Sox4* 3′UTR in NPCs. We performed a miR pull-down in NPCs transfected with biotinylated miR-129-5p or scramble miR-control and assessed by qPCR the abundance of *Sox4* mRNA. We were able to detect a two-fold increase in the abundance of *Sox4* mRNA compared to the scramble miR (Figure 5b). This confirmed that miR-129-5p binds to *Sox4* 3′UTR in NPCs.

Then, we wanted to evaluate if the overexpression of miR-129-5p in NPCs in MC conditions decreased the protein levels of SOX4. To assess that, we transfected a miR-129-5p mimic or a scramble miR and evaluated SOX4 protein levels by Western blot. We found a ~35% reduction in SOX4 protein levels when we transfected miR-129-5p compared to scramble miR (Figure 5c,d).

Conversely, we sought to determine if blocking miR-129-5p was sufficient to restore SOX4 protein levels in our NPC model, in LC conditions. To accomplish this, we transfected NPCs with either miR-129-5p inhibitor or scramble miR inhibitor and treated the cells with MC or LC. We found that NPCs treated with LC status and transfected with miR-129-5p inhibitor resulted in an increase in SOX4 protein levels when compared to scramble inhibitor and MC (Figure 5e,f).

All taken together, these results demonstrated that choline regulates SOX4 expression via miR-129-5p in NPC.

### 3.7. Low Choline Reduces the Trimethylated Histone H3 at Lysine 27 (H3K27me3) in Fetal Cortices

Next, we asked how choline directly, or indirectly, regulates miR-129-5p. Choline can be used as a methyl donor, through S-adenosylmethionine [51], and we previously demonstrated that LC alters the methylation potential in LC embryonic brains [19]. Hence, we hypothesized that a reduction in the methylation potential, via LC, in NPCs reduces DNA methylation in the transcriptional regulatory elements of miR-129-5p. To test this hypothesis, we performed targeted next-generation bisulfite sequencing in NPCs for the miR-129 gene. In mice, miR-129-5p was transcribed from two genes—miR-129-1 and miR-129-2, located in mouse chromosome 2 and chromosome 6 [52]. We evaluated DNA methylation in both locations; however, we did not find significant differences when comparing MC vs. LC (Tables S3 and S4).

**Figure 6:**
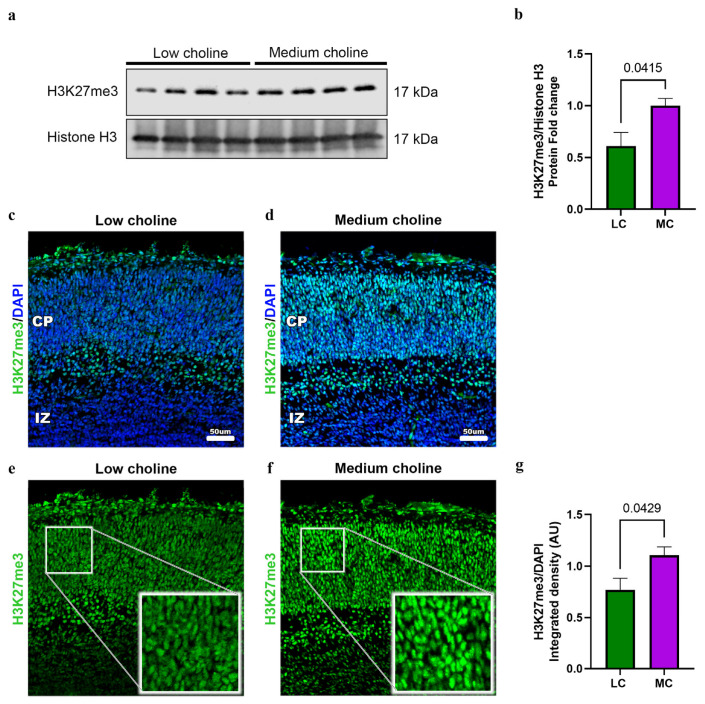
Low choline availability reduces global H3K27me in fetal brains. Dams were placed on either an LC or MC diet from E11.5 to E17.5. (**a**) Cerebral cortex of E17.5 embryos were dissociated to extract total histones. Cell lysates were analyzed by Western blot with antibodies to H3K27me3 and Total Histone 3 (H3). We observed a reduction in the protein levels of H3K27me3 in LC brains. (**b**) Quantifications of protein levels are normalized to H3 and presented as fold change. (*n* = 4 dams; 1 pup per dam) (*p* = 0.0415). (**c**) Representative immunostaining from an E17.5 cerebral cortex exposed to low choline or (**d**) medium choline. Low-choline brains exhibit reduced levels of H3K27me3 when compared to medium choline. (**e**) H3K27me3 staining in the CP in low-choline brains (**f**) H3K27me3 staining in the CP in medium choline brains. Cerebral cortex exposed to LC availability have lower H3K27me3 expression compared to MC. (**g**) Quantification of H3K27me protein levels was performed in LC and MC by detection of integrated density from immunofluorescence, levels were normalized with DAPI. (*n* = 4–5 dams per condition, 1 pup per dam) (*p* = 0.0429). Data are mean ± SEM. All data were tested for normality by Brown–Forsythe test and statistical analysis was performed using an unpaired *t*-test.

Our alternative hypothesis was that a reduction in the methylation potential was impacting histone methylation. Post-transcriptional modifications of histones during neural development are fundamental to the regulation of transcription and the switch between the maintenance of self-renewal and the differentiation into other cell types [53]. In the cerebral cortex, EZH2 is key for controlling cortical progenitor cell programming through the regulation of the repressive mark of the trimethylated histone H3 at lysine 27 (H3K27me3) [28]. As mentioned above, we observed a reduction in the protein levels of EZH2. This led us to investigate if the protein levels of H3K27me3 in cortices from embryonic brains were different in LC or MC conditions. We observed in LC a 50% reduction in the total levels of H2K27me3 (Figure 6a,b). To confirm that these changes were specific for H3K27me3, we assessed the total levels of the trimethylated histone H3 at lysine 9 (H3K9me3) and found no significant changes (Figure S1B,C).

Then, to further confirm our results, we performed immunostainings to detect levels of H2K27me3 in the developing cortex of embryos from LC and MC. We found a ~40% reduction in H3K27me3 in the developing cortical plate (CP) (Figure 6c–g) after quantifying integrated density normalized by DAPI (Figure 6g). Moreover, we confirmed that global transcription is not affected, since levels of phosphorylated RNA pol II remain the same under MC vs. LC conditions (Figure S2A–C).

Overall, these results show that low choline decreases the levels of H3K27me3 in the developing brain.

## 4. Discussion

The crucial role of choline in brain development is firmly established [1,54,55]. However, the answers around the molecular mechanism by which maternal choline impacts the developing fetal brain are still developing. Here, we report for the first time that low choline availability reduces the transcription factor SOX4 during neurogenesis.

SOX4 has critical developmental functions such as skeletal and heart development; a lack of SOX4 is lethal in utero [56,57]. Additionally, the postnatal deletion of SOX4 in mice causes sympathetic nervous system defects [58]. We show here that low choline availability reduces SOX4 protein levels in the cortex and that its mRNA remains unchanged. Furthermore, we found a whole-cell reduction in SOX4 protein; however, this reduction was greater in the nucleus compared to that of the cytoplasm. This cell compartmentalization difference in SOX4 is relevant considering the nucleus is where it functions as a transcription factor. SOX4 has many transcriptional targets, including EZH2 [29]. Together, SOX4 and EZH2 act as a molecular switch that facilitates the transition of NPCs and IPCs from progenitor cells to differentiated neurons [25,28,29]. In our study, we found that the reduction in SOX4 in the nucleus subsequently led to the reduction in *Ezh2* mRNA and protein, demonstrating that SOX4 alters EZH2 at the transcriptional level depending on choline status. The reduction in both SOX4 and EZH2 in NPCs, under LC conditions, suggests changes to NPC cell fate. EZH2 is required for cell identity through depositing the histone mark H3K27me3, which controls the acquisition of neural fate [59,60]. In human neural progenitor cells, EZH2 is required for proliferation and neural fate decision [61]. SOX4 and EZH2 aberrant expression in LC NPCs potentially helps to explain, in part, earlier observations made by our lab. We showed that LC availability reduces the number of late-born neurons, at embryonic day 17.5 (E17.5), resulting in altered cortical layering [14]. The observed phenotype is caused by nutrient stress, specifically low choline, and we speculate that a compensating mechanism is turned on to modify the cell fate decision in the developing brain.

Transcriptional regulation is carefully orchestrated by synthesis and degradation to maintain optimal levels. As mentioned before, mechanisms to maintain adequate SOX4 protein levels are mediated, in part, through its binding with SDCBP to prevent proteasomal degradation [50]. However, we found no changes in *Sdcbp* mRNA or protein levels in NPCs from MC vs. LC. Another potential mechanism for SOX4 degradation included choline interfering with protein synthesis, and we did not find significant differences between LC and MC protein synthesis levels. This informed us that the mechanism by which SOX4 protein levels are regulated by choline are post-transcriptional and specifically miR-mediated.

miRs are small noncoding RNAs that regulate gene expression at a post-transcriptional level. Specifically in the brain, miRs help to downregulate multiple genes to ensure proper protein levels and function [62]. We previously showed that miR-129-5p is upregulated in LC and targets *Egfr*, reducing NPC self-renewal capacity [19]. miRs can target multiple genes, and miR-129 also targets Fragile X mental Retardation gene 1 (*Fmr1*) and influences neuronal migration in the developing cortex, in a fragile X model [43]. Previously, we showed that an overexpression of miR-129-5p, in vivo, under MC conditions alters neuronal migration, and that inhibiting miR-129-5p in vivo, under LC conditions, rescued the proliferation phenotype.

Here, we demonstrate that choline regulates SOX4 through miR-129-5p. We determined this thorough miR-129-5p gain of function and loss of function and its impact on SOX4 protein levels. This is consistent with findings in other models, including a breast cancer cell line and a melanoma cell line where miR-129-5p targets SOX4 [24,63]. In both models, it was demonstrated that the expression of miR-129-5p was regulated, in part, by promoter H3K27me3 methylation, mediated by EZH2 [24,63]. This seems highly likely to also be the mechanism by which miR-129-5p is upregulated in NPCs under an LC status, considering the lack of changes in CpG island methylation around the promoter of miR-129-5p. Previously, we only evaluated H3K27me3 in NPCs in vitro and did not find significant changes; however, since this histone mark is part of an epigenetic switch towards differentiation, the analysis in the whole cortex, with different cell types, is a better approach. Additionally, there is enough evidence of how choline decreases the methylation potential in the brain [19,64,65]. We speculate that choline epigenetically regulates miR-129-5p by SOX4/EZH2/H3K27me3 by a feedback loop.

One of the limitations of this study is the reliance on indirect evidence that links H3K27me3 as a regulator of miR-129-5p. Our future directions are focused on elucidating the mechanism by which this histone mark directly impacts the expression of miR-129-5p and, potentially, other genes. This may provide a comprehensive understanding of how choline availability regulates gene expression involving transcription factors such as SOX4 and epigenetic modifications, specifically H3K27me3.

Our observations confirmed that the hippocampus and cerebral cortex development depend on choline availability. Here, for the first time, we are linking choline availability as a mechanism to regulate SOX4 expression.

Altogether, our findings are of interest to human health. Our preclinical data provide evidence of the importance of adequate choline intake for cerebral cortex development. According to the latest NHANES survey in the US, pregnant women are consuming below the recommended choline intake (recommended intake of 425 mg/day) [9]. Additionally, LC intake during pregnancy correlates with poor visual memory in children at 7 years old [66], showing that in-utero LC availability has long-lasting effects. A recent meta-analysis showed that women consuming an LC diet increased up to 2.5 times the risk of neural tube defects [67]. Inadequate choline consumption is typical in women of reproductive age, and efforts must be made to rethink recommendations on choline intake to ensure proper brain development and function. Finally, choline availability may have an outsized effect on perturbed fetal brain development, which may lead to the worsening of neurodevelopmental disorders.

## Data Availability

Data included in article/supplementary material/referenced in article.

## Supplementary Materials

The following supporting information can be downloaded at: https://www.mdpi.com/article/10.3390/nu15122774/s1, Table S1: Diet composition; Table S2: Primer sequences; Figure S1: In silico prediction of miR-129-5p binding sites within *Sox4* 3′UTR in red and global H3K9me3 in embryonic cortices; Figure S2: Choline availability does not alter global transcription in the developing brain. Table S3: Analysis of CpG methylation of the Mir129-1 gene does not exhibit changes when comparing LC vs. MC; Table S4: Analysis of CpG methylation of the Mir129-2 gene does not exhibit changes when comparing LC vs. MC.
