# Supplementary material for: Choline Regulates SOX4 through miR-129-5p and Modifies H3K27me3 in the Developing Cortex
Source: Nutrients. 2023 Jun 16;15(12):2774. doi: 10.3390/nu15122774 (PMC10304412; doi:10.3390/nu15122774)
Supplement: Supplementary file 1 [file nutrients-15-02774-s001.zip › nutrients-2438266-supplementary.pdf]

1. Supplementary Materials

Product number	Low Choline D16040705		Medium Choline D16040703		High Choline D16040706	
	gm%	kcal%	gm%	kcal%	gm%	kcal%
Protein	21	20	21	20	21	21
Carbohydrate	64	64	64	64	63	64
Fat	7	16	7	16	7	16
Total		100		100		100
kcal/gm	4		4		4	
**Ingredient**	**gm**	**kcal**	**gm**	**kcal**	**gm**	**kcal**
Casein	0	0	0	0	0	0
Isolated Soy Protein	200	800	200	800	200	800
L-Cystine	5.08	20	2.54	10	2.54	10
L-Methionine			2.54	10	2.54	10
Corn Starch	397.906	1592	396.506	1586	391.606	1566
Maltodextrin 10	132	528	132	528	132	528
Sucorse	100	400	100	400	100	400
Cellulose, BW200	50	0	50	0	50	0
Soybean Oil	70	630	70	630	70	630
t-Butylhydroquinone	0.014	0	0.014	0	0.014	0
Mineral Mix S10022G	35	0	35	0	35	0
Vitamin Mix V10037	10	40	10	40	10	40
Choline Chloride	0	0	1.4	0	6.3	0
Choline Bitartrate	0	0	0	0	0	0
FD&C Blue Dye #1	0	0	0	0	0	0
FD&C Yellow Dye #5	0	0	0	0	0	0
FD&C Red Dye #40	0	0	0	0	0	0
**Total**	**1000**	**4010**	**1000**	**4004**	**1000**	**3985**

Table S1. Diet composition for low, medium, and high choline.

Primer	Sequence
B-Actin (PrimerBank: ID6671509a1)	FWD: GGCTGTATTCCCCTCCATCG
	REV: CCAGTTGGTAACAATGCCATGT
Ezh2 (PrimerBank: ID26353604a1)	FWD: AGTGACTTGGATTTTCCAGCAC
	REV: AATTCTGTTGTAAGGGCGACC
Sdcbp (PrimerBank: ID7949150a1)	FWD: CACCAGCACAAGGGTTGGTAG
	REV: GCCTGGACGAGTTGAACAAAT
Sox4 (PrimerBank: ID6678073a1)	FWD: CGGCTGCATCGTTCTCTCC
	REV: GGTAGACGTGCTTCACTTTCTTG
Tbt (PrimerBank: ID8850234a1)	FWD: AGAACAATCCAGACTAGCAGCA
	REV: GGGAACTTCACATCACAGCTC

Table S2. Primer sequences used RT-PCR Analysis.

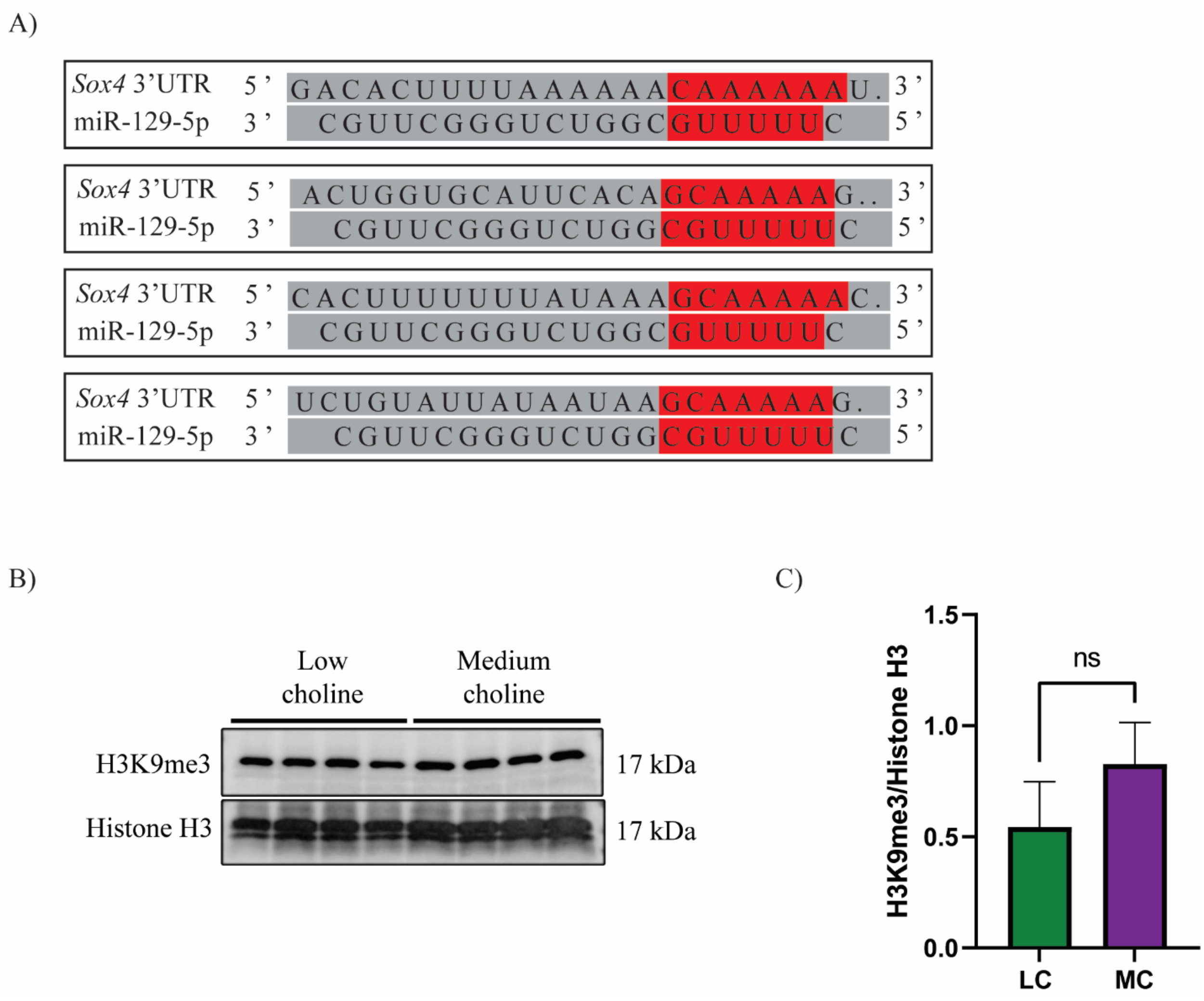

Figure S1. A) In silico prediction of miR-129-5p binding sites withing *Sox3* 3′UTR in red. Dams were placed on either a LC or MC diet from E11.5 to E17.5. B) Cerebral cortex of E17.5 embryos were dissociated to extract total histones. Cell lysates were analyzed by western blot with antibodies to H3K9me3 and Total Histone 3 (H3). We did not observe changes in the global protein levels of H3K9me3 when comparing LC vs MC. C) Quantifications of protein levels are normalized to H3 and presented as fold change. (n= 4 dams; 1 pup per dam) (ns: not significant; p=0.3639). Data are mean ± SEM. Data was tested for normality by Brown-Forsythe test and statistical analysis was performed using an unpaired t-test.

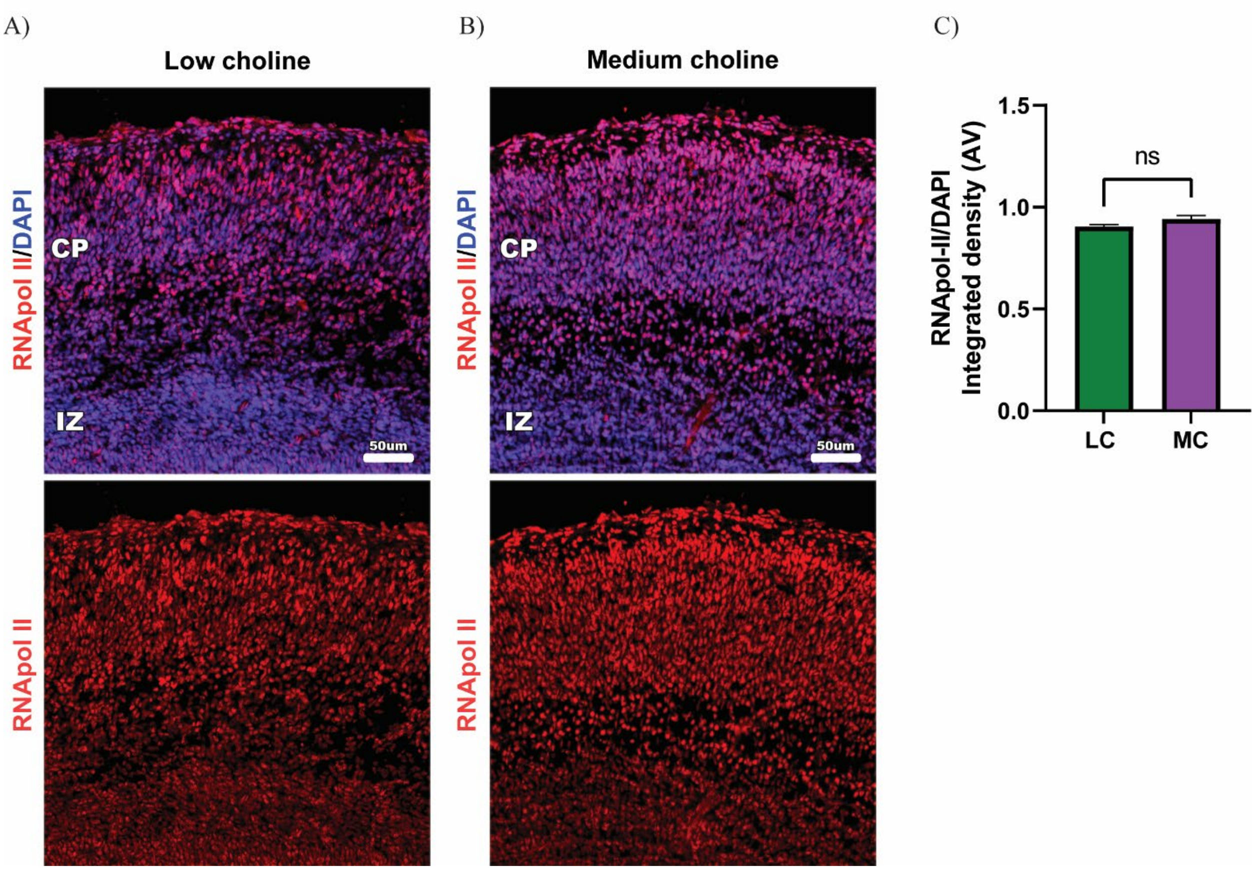

Figure S2. Choline availability does not alter global transcription in the developing brain. Dams were placed on either a LC or MC diet from E11.5 to E17.5. A-B) Representative immunostaining from an E17.5 cerebral cortex exposed to LC of MC. These brains do not exhibit changes in global transcription. C) Quantification of RNA pol ll protein levels was performed in LC and MC by detection of immunofluorescence (n=4-5 dams per condition, 1 pup per dam) (ns: not significant; p=0.0974). Data are mean ± SEM. All data was tested for normality by Brown-Forsythe test and statistical analysis was performed using an unpaired t-test.

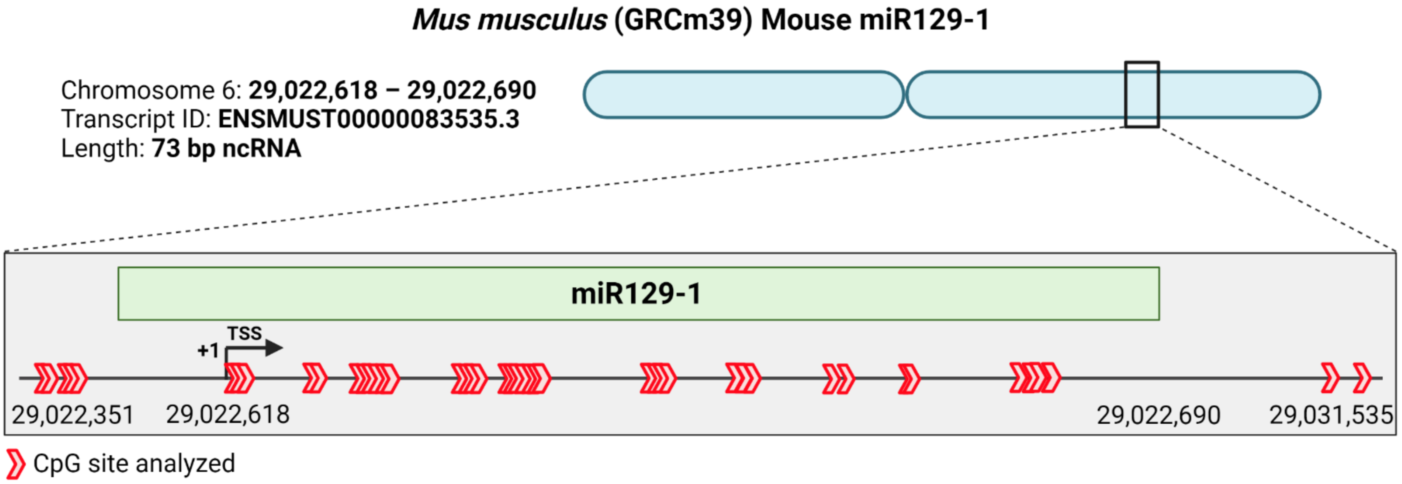

Location	From TSS (ENSMUST00000083535.3)	CpGs	GRCm39	CpG location	Mean % methylation (n=4 per group)		p value
					LC	MC	
5-Upstream	-267 to -189	5	Chr6:29022351	-267	89.43	88.88	0.1056
			Chr6:29022370	-248	92.50	93.23	0.2257
			Chr6:29022405	-213	89.25	89.80	0.6929
			Chr6:29022414	-204	63.68	62.78	0.6460
			Chr6:29022429	-189	90.45	89.05	0.4653
Exon 1	+13 to +35	3	Chr6:29022630	+13	91.05	89.78	0.6342
			Chr6:29022652	+35	85.10	85.85	0.8048
			Chr6:29022691	+74	95.33	95.45	0.9551
3-Downstream	+664 to +687	2	Chr6:29023281	+664	68.85	69.50	0.3143
			Chr6:29023304	+687	61.55	63.23	0.8857
3-Downstream	+867 to +985	6	Chr6:29023484	+867	7.37	12.53	0.0559
			Chr6:29023501	+884	19.45	14.03	0.4119
			Chr6:29023519	+902	42.95	40.75	0.6063
			Chr6:29023542	+925	42.20	36.35	0.2330
			Chr6:29023567	+950	44.55	48.93	0.4483
			Chr6:29023602	+985	22.08	19.40	0.7868
3-Downstream	+1400 to +1447	4	Chr6:29024017	+1400	40.38	40.78	0.7692
			Chr6:29024034	+1417	26.83	27.28	0.8253
			Chr6:29024037	+1420	24.23	23.60	0.5906
			Chr6:29024064	+1447	33.00	31.70	0.3388
3-Downstream	+1687 to +1741	7	Chr6:29024304	+1687	1.65	2.22	0.4041
			Chr6:29024318	+1701	0.22	0.90	0.2289
			Chr6:29024328	+1711	0.40	0.27	0.6803
			Chr6:29024335	+1718	0.50	0.55	0.9013
			Chr6:29024348	+1731	0.22	0.65	0.2597
			Chr6:29024354	+1737	0.00	0.57	0.1871
			Chr6:29024358	+1741	0.57	0.25	0.2838
3-Downstream	+2441 to +2502	4	Chr6:29025058	+2441	85.06	84.75	0.6905
			Chr6:29025091	+2474	84.85	82.63	0.3429

			Chr6:29025112	+2495	91.90	92.15	0.7993
			Chr6:29025119	+2502	93.20	91.68	0.2298
3-Downstream	+3683 to +3724	3	Chr6:29026300	+3683	78.30	78.25	0.9498
			Chr6:29026305	+3688	83.58	83.00	0.1046
			Chr6:29026341	+3724	41.63	40.35	0.4321
3-Downstream	+4280 to +4334	2	Chr6:29026897	+4280	71.95	69.08	0.2312
			Chr6:29026951	+4334	21.75	21.18	0.2526
3-Downstream	+4552 to +4576	2	Chr6:29027169	+4552	64.23	64.08	0.8915
			Chr6:29027193	+4576	61.95	60.38	0.4875
3-Downstream	+6350 to +6444	4	Chr6:29028967	+6350	92.70	92.13	0.6403
			Chr6:29028996	+6379	93.98	92.85	0.5207
			Chr6:29029033	+6416	93.60	91.10	0.046*
			Chr6:29029061	+6444	79.78	79.28	0.6875
3-Downstream	+8832 to +8918	4	Chr6:29031449	+8832	1.75	1.72	0.9799
			Chr6:29031457	+8840	8.20	0.18	0.9890
			Chr6:29031463	+8846	13.70	13.65	0.9650
			Chr6:29031535	+8918	9.57	10.48	0.6724

Table S3. Analysis of CpG methylation of the Mir129-1 gene does not exhibit changes when comparing LC vs. MC. Cortical NPCs were cultured in either LC (5µM) or MC (70µM) for 48 hours and subjected to bisulfite conversion to evaluate methylation. A total of 46 methylation sites in the sequence 29,022,618 to 29,022,690 in chromosome 6 were analyzed, 5 CpGs 5'Downstream, 3 within Exon-1 and 38 in the 3' Downstream region. The CpG site Chr6:29029033 (+6416) is statistically significant (p=0.046, t-test); however, the overall percentage of methylation between the two groups does not represent a significant change.

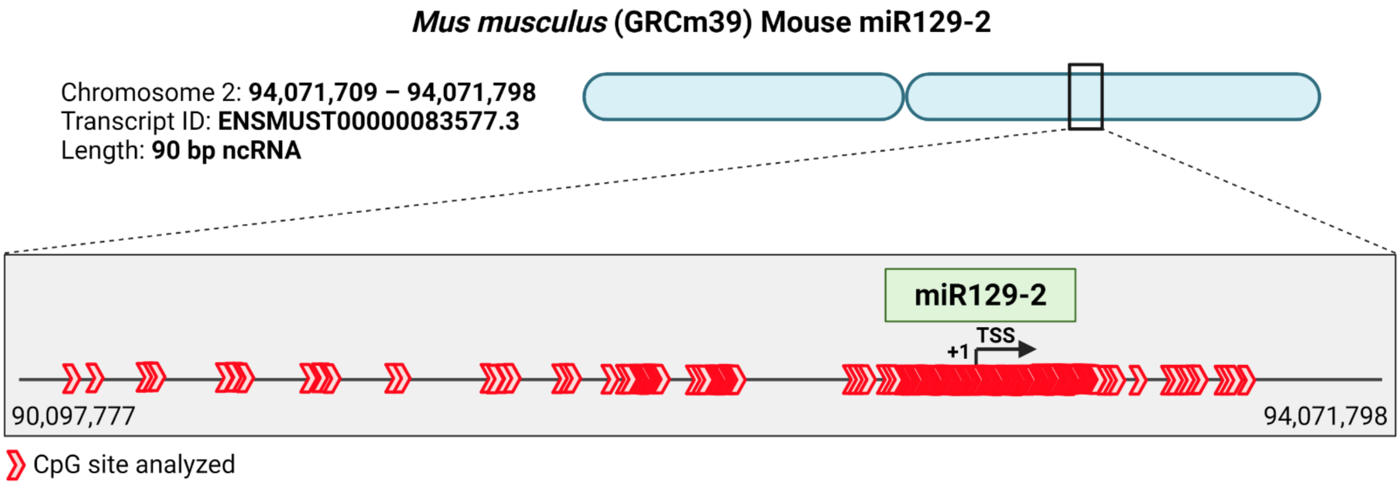

Location	From TSS (ENSMUST00000129661.3)	CpGs	GRCm39	CpG location	Mean % methylation (n=4 per group)		p value
					LC	MC	
5-Upstream	-2403 to -2364	2	Chr2:94097777	-2403	6.75	6.77	0.9645
			Chr2:94097738	-2364	13.90	13.33	0.7056
5-Upstream	-1996 to -1931	3	Chr2:94097777	-1996	58.87	53.50	0.1328
			Chr2:94097738	-1968	35.37	32.80	0.7140
			Chr2:94097370	-1931	23.30	23.10	0.9754
5-Upstream	-149 to -56	4	Chr2:94097342	-149	1.20	0.60	0.4825
			Chr2:94097305	-93	1.25	0.20	0.3369
			Chr2:94095523	-86	1.07	0.23	0.3159
			Chr2:94095467	-56	2.02	0.48	0.1249
Intron 1	+1283 to +1350	7	Chr2:94095460	+1283	1.70	1.40	0.8795
			Chr2:94095430	+1297	2.27	1.27	0.5891
			Chr2:94094092	+1303	6.37	0.95	0.3651
			Chr2:94094078	+1305	1.62	0.67	0.4411
			Chr2:94094072	+1311	1.97	0.60	0.3648
			Chr2:94094070	+1317	1.87	1.10	0.5569
			Chr2:94094064	+1350	2.10	0.65	0.3590
5-Upstream	-6131 to -6067	3	Chr2:94094058	-6131	4.05	6.07	0.1455
			Chr2:94094025	-6071	20.20	21.60	0.6370
			Chr2:94077929	-6067	5.33	9.26	0.2192
5-Upstream	-5674 to -5647	4	Chr2:94077869	-5674	0.80	0.75	0.9425
			Chr2:94077865	-5669	1.35	1.05	0.6447
			Chr2:94077472	-5662	1.02	1.07	0.9478
			Chr2:94077467	-5647	1.75	1.35	0.3112
5-Upstream	-4942 to -4863	4	Chr2:94077460	-4942	54.85	49.95	0.3138
			Chr2:94077445	-4903	53.30	45.15	0.1925
			Chr2:94076740	-4871	11.58	14.03	0.1405
			Chr2:94076701	-4863	33.53	29.65	0.1885
5-Upstream	-4150 to -4050	4	Chr2:94076669	-4150	4.60	4.20	0.5501
			Chr2:94076661	-4101	3.27	2.22	0.1133

			Chr2:94075948	-4072	4.67	4.00	0.0825
			Chr2:94075899	-4051	3.15	2.25	0.2507
5-Upstream	-3845 to -3816	3	Chr2:94075870	-3845	66.48	65.10	0.2854
			Chr2:94075849	-3841	91.65	91.65	0.9999
			Chr2:94075643	-3816	51.78	53.15	0.2598
5-Upstream	-3134 to -3043	5	Chr2:94075639	-3134	96.80	96.03	0.2714
			Chr2:94075614	-3117	22.55	23.68	0.2608
			Chr2:94074932	-3086	60.45	61.58	0.3061
			Chr2:94074915	-3048	58.80	58.43	0.1764
			Chr2:94074884	-3043	55.45	52.28	0.0600
5-Upstream	-2619 to -2594	3	Chr2:94074846	-2619	12.45	11.38	0.2855
			Chr2:94074841	-2611	55.50	17.70	0.0518
			Chr2:94074417	-2594	56.95	55.00	0.0838
5-Upstream	-415 to -379	3	Chr2:94074409	-415	10.75	9.00	0.1085
			Chr2:94074392	-399	9.07	9.82	0.7053
			Chr2:94072213	-379	4.00	3.75	0.6711
5-Upstream	-316 to -199	7	Chr2:94072197	-316	3.92	3.97	0.9690
			Chr2:94072177	-295	7.65	7.25	0.8452
			Chr2:94072114	-265	1.25	0.72	0.4677
			Chr2:94072093	-252	3.20	2.82	0.8301
			Chr2:94072063	-238	5.82	6.10	0.8915
			Chr2:94072050	-220	3.12	3.50	0.7670
			Chr2:94072036	-199	2.57	1.20	0.2274
5-Upstream	-160 to -78	12	Chr2:94072018	-160	1.75	1.82	0.7045
			Chr2:94071997	-158	0.90	1.22	0.2838
			Chr2:94071958	-144	2.50	2.27	0.2665
			Chr2:94071956	-141	1.60	1.32	0.6697
			Chr2:94071942	-139	1.57	1.32	0.3198
			Chr2:94071939	-118	0.92	1.22	0.6283
			Chr2:94071937	-116	1.32	1.62	0.1343
			Chr2:94071916	-108	2.22	2.77	0.2810
			Chr2:94071914	-106	2.07	2.20	0.8603
			Chr2:94071906	-103	2.62	2.52	0.8679
			Chr2:94071904	-84	2.57	2.52	0.9096
			Chr2:94071901	-78	2.22	3.65	0.0582
5-Upstream	-49 to -9	11	Chr2:94071882	-49	3.32	3.05	0.5419
			Chr2:94071876	-46	3.40	3.35	0.9365
			Chr2:94071847	-43	1.85	1.65	0.6653
			Chr2:94071844	-40	2.52	2.02	0.3868
			Chr2:94071841	-37	2.45	2.05	0.2147
			Chr2:94071838	-34	2.75	2.37	0.4553
			Chr2:94071835	-31	2.92	3.05	0.1250
			Chr2:94071832	-25	0.407	3.35	0.1881
			Chr2:94071829	-22	4.42	3.57	0.2695
			Chr2:94071823	-17	3.92	3.75	0.7711
			Chr2:94071820	-9	2.30	2.30	0.9999
Exon 1	+8 to +22	3	Chr2:94071815	+8	2.75	2.17	0.1387

			Chr2:94071807	+10	1.52	1.12	0.4610
			Chr2:94071791	+22	2.17	1.82	0.5044
Exon 1	+54 to +89	4	Chr2:94071789	+54	9.00	7.67	0.6179
			Chr2:94071777	+80	6.17	6.15	0.9933
			Chr2:94071745	+82	7.10	6.90	0.9537
			Chr2:94071719	+89	7.75	5.85	0.3557
3-Downstream	+91 to +289	21	Chr2:94071717	+91	8.02	10.15	0.4493
			Chr2:94071710	+95	5.02	6.05	0.7139
			Chr2:94071708	+98	9.65	6.42	0.2778
			Chr2:94071704	+101	7.55	7.02	0.8615
			Chr2:94071701	+119	5.62	4.22	0.6271
			Chr2:94071698	+123	6.17	5.75	0.9056
			Chr2:94071680	+126	7.57	5.65	0.3927
			Chr2:94071676	+143	6.90	2.67	**0.009***
			Chr2:94071673	+148	5.72	3.12	0.3041
			Chr2:94071656	+158	5.30	6.22	0.7088
			Chr2:94071651	+182	4.22	3.50	0.8410
			Chr2:94071608	+224	13.73	9.16	0.0879
			Chr2:94071575	+231	7.83	4.70	0.2943
			Chr2:94071573	+233	10.52	4.23	0.1265
			Chr2:94071568	+249	20.60	17.37	0.4566
			Chr2:94071566	+257	30.27	23.23	0.1994
			Chr2:94071550	+271	17.90	15.97	0.8298
			Chr2:94071542	+289	3.27	1.50	**0.009***
3-Downstream	+317 to +389	5	Chr2:94071528	+317	14.73	15.13	0.6782
			Chr2:94071510	+324	30.78	30.28	0.2715
			Chr2:94071482	+328	31.43	32.13	0.7552
			Chr2:94071475	+338	26.98	27.43	0.8171
			Chr2:94071471	+389	13.58	11.58	0.2438
3-Downstream	+567 to +646	6	Chr2:94071461	+567	50.68	51.50	0.7857
			Chr2:94071410	+587	39.38	39.98	0.8516
			Chr2:94071232	+600	90.58	90.25	0.6806
			Chr2:94071212	+629	83.48	82.20	0.5615
			Chr2:94071199	+637	88.23	87.98	0.8377
			Chr2:94071170	+646	90.33	88.73	0.1789
3-Downstream	+799 to +889	5	Chr2:94071162	+799	87.90	89.95	0.1656
			Chr2:94071153	+818	39.33	40.63	0.4259
			Chr2:94071000	+820	54.73	54.80	0.9832
			Chr2:94070981	+837	83.30	83.13	0.8929
			Chr2:94070979	+889	78.35	76.80	0.2929

Table S4. Analysis of CpG methylation of the Mir129-2 gene does not exhibit changes when comparing LC vs. MC. Cortical NPCs were cultured in either LC (5μM) or MC (70μM) for 48 hours and subjected to bisulfite conversion to evaluate methylation. A total of 119 methylation sites in the sequence 29,022,618 to 29,022,690 in chromosome 2 were analyzed, 68 CpGs 5'Downstream, 7 within Intron-1, 7 in Exon-1 and 37 in the 3'Downstream region. The CpGs sites Chr2:94071676 (+143) and Chr2:94071542 (+289) were

statistically significant ($p=0.009$, t-test); however, the percentage of methylation between the two groups does not represent a significant change.
